# Effects of Fineness and Morphology of Quartz in Siliceous Limestone on the Calcination Process and Quality of Cement Clinker

**DOI:** 10.3390/ma17143601

**Published:** 2024-07-21

**Authors:** Donggen Nie, Wei Li, Lilan Xie, Min Deng, Hao Ding, Kaiwei Liu

**Affiliations:** 1 College of Material Science and Engineering, Nanjing Tech University, Nanjing 211816, China; 2 Anhui Province Key Laboratory of Advanced Building Materials, Anhui Jianzhu University, Hefei 230000, China; 3 School of Materials and Architectural Engineering, Guizhou Normal University, Guiyang 550001, China; 4 Hefei Cement Research and Design Institute Corporation Ltd., Hefei 230000, China

**Keywords:** siliceous limestone, clinker, quartz, microstructure, fineness

## Abstract

With the increasing depletion of high-quality raw materials, siliceous limestone, sandstone and other hard-to-burn raw materials containing crystalline SiO_2_ are gradually being used to produce clinker. This study investigates the influence of the quartz content and particle size in siliceous limestone on the calcination process and the resultant quality of cement clinker. Two different siliceous limestones were grinded to different fineness, and calcinated with some other materials. The content of the clinkers was analyzed with the XRD–Rietveld method and the microstructure of the clinkers was observed with laser scanning confocal microscopy (LSCM) and field emission scanning electron microscopy (FESEM). Three key outcomes of this study provide new insights on the use of siliceous limestone in cement production, namely that (i) reducing the fineness values of siliceous limestone from 15% to 0% of residue on a 0.08 mm sieve decreases the quantity of these larger quartz particles, resulting in an increase in C_3_S content by up to 8% and an increase in 28d compressive strength by up to 4.4 Mpa, which is 62.30 Mpa; (ii) the morphology of quartz—either as chert nodules or single crystals—affects the microstructure of C_2_S clusters in clinker, finding that chert nodules result in clusters with more intermediate phases, whereas large single crystals lead to denser clusters; (iii) the sufficient fineness values of siliceous limestone SL1 and SL2 are 5% and 7% of residue on a 0.08 mm sieve, respectively, which can produce a clinker with a 28d compressive strength greater than 60 Mpa, indicating that for different kinds of quartz in siliceous limestone, there is an optimum grinding solution that can achieve a balance between clinker quality and energy consumption without having to grind siliceous limestone to very fine grades.

## 1. Introduction

Carbon emission reduction in the cement industry is a key and difficult issue in the implementation of the “double carbon” goal. SiO_2_ is the main component of cement raw meal [1], and the crystalline SiO_2_ in raw meal has a decisive influence on the energy consumption and clinker quality during the process of calcination. With the increasing depletion of high-quality raw meal, siliceous limestone, sandstone, shale and other hard-to-burn raw meal containing crystalline quartz are gradually being used to produce cement clinker. Studies have shown that the large-size quartz in the raw meal are weak to abrasion, and may influence the burnability of raw meal and the resultant quality of cement clinker [2,3]. The question of how to solve the problem of clinker performance degradation caused by large-size quartz is of great importance to improve clinker strength and reduce the energy consumption of clinker production.

SiO_2_, as the main component of Portland cement raw meal, mainly exists in the form of quartz minerals and silicate minerals in the raw meal. Quartz is a stable tetrahedral Si-O structure, with a melting point higher than the clinker calcination temperature, making it difficult to depolymerize into more reactive [SiO_4_]^4−^ structure; hence, its ability to combine with CaO to form C_2_S and C_3_S is poor compared to silicate minerals, which is unfavorable for clinker calcination [4]. Quartz undergoes polymorphic transformations at different temperatures, but its tetrahedral Si-O structure does not change; thus, its impact on clinker calcination is still noticeable. However, research has found that the reactivity of different polymorphs of quartz crystals with CaO does show some differences, typically increasing in the following order: α-quartz < chalcedony < α-tridymite < α-cristobalite < amorphous SiO_2_ [5,6]. Besides, the particle size and content of quartz are other key factors affecting the calcination of raw meal [7,8,9,10,11].

Currently, it is widely believed that the fineness of quartz particles in raw meal significantly affects the calcination of clinker [7,8,12,13,14,15,16]. Quartz particles mainly consist of quartz crystals of various shapes and sizes. Fundal and Christensen et al. [7,17,18] have proposed that quartz particle sizes exceeding 45 µm can adversely affect clinker calcination, whereas quartz particles smaller than 45 µm do not impact the reactivity of raw meal. Jiang et al. [19] investigated the distribution of quartz particles in different sandstone powders after grinding and their relationship with the content of f-CaO in the clinker. They found that the coarse quartz particle (125–160 µm) content has a correlation of about 95% with the content of f-CaO, while quartz particles smaller than 30 µm only have a correlation of about 50% with the content of f-CaO, indicating that the larger the quartz particles, the greater the impact on the content of f-CaO. Zhang [9] has used quartz-based sandstone of different levels of fineness to produce clinker, studying the effect of quartz particle fineness on the content of f-CaO in the clinker. It was pointed out that when the sieve residue of quartz particles exceeds 0.89% at 80 µm, the content of f-CaO will exceed 1.5%. It is generally believed that the impact of quartz crystals on calcination is that quartz crystals affect the formation of belite, thereby affecting calcination and leading to a decline in quality [15]. However, some studies have pointed out that coarse quartz particles react with CaO to form different belite clusters. The surface of these belite cluster is dense, which hinders the reaction between belite and CaO, thus affecting the formation of alite [20,21] and leading to an increase in the content of f-CaO [22,23,24].

The current research on the impact of quartz fineness on calcination is primarily focused on quartz particles, which are mixtures composed of quartz crystals of various shapes and sizes, and other minerals. This complex and diverse structure cannot accurately reflect the reaction processes of different quartz crystals and its influence on the calcination of clinker [25]. Therefore, further studies are needed, and so the purpose of this paper is to investigate the effect of the fineness and morphology of quartz in siliceous limestones on the calcination process and quality of the clinker. Two siliceous limestones with different morphologies of quartz from China were used. The internal standard methods of X-ray diffraction (XRD) and polarizing microscope analysis were chosen to analyze the distribution of quartz in raw meal. The XRD–Rietveld method [26] was chosen to determine the quantity of clinker minerals, and laser scanning confocal microscopy (LSCM) was used to analyze the microstructure of the clinker. Field emission scanning electron microscopy (FESEM) equipped with EDS was used to analyze the elemental distribution of clinker minerals. This investigation is anticipated to advance the knowledge on quartz’s role in clinker calcination, and offer practical insights for improving the efficiency of clinker calcination using siliceous limestone.

## 2. Materials and Methods

### 2.1. Materials

**Figure 1:**
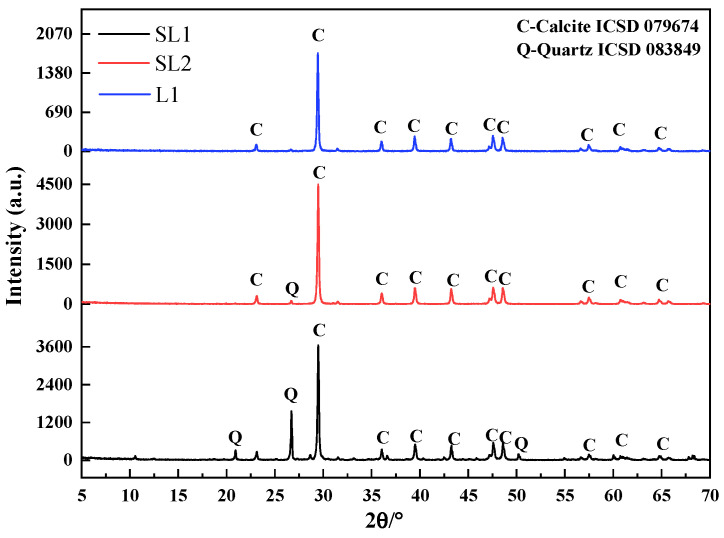
X-ray diffraction patterns of siliceous limestone SL1, SL2 and limestone L1.

Siliceous limestones SL1 and SL2, derived from Hongshi Cement Company located in Sanming, Fujian, China and Southwest Cement Company located in Guiyang, Guizhou, China, were used. Limestone L1, sandstone S1, aluminum corrective material FA, iron corrective material IS and coal derived from South Cement Company located in Hefei, Anhui, China were used. Table 1 shows the chemical compositions of the raw materials analyzed according to the standard GB/T 176-2017 [27], with Thermo ARL 9900 X-ray fluorescence (XRF) (Waltham, America). Siliceous limestones SL1 and SL2 contained 27.02% and 6.48% SiO_2_, respectively. While SL1, SL2 and limestone L1 contained 37.00%, 50.91% and 53.84% CaO, respectively. Siliceous sandstone S1 contained 73.08% SiO_2_. Aluminum corrective material FA contained 24.94% Al_2_O_3_, and iron corrective material IS contained 45.57% Fe_2_O_3_. Figure 1 shows the XRD patterns of three types of limestone determined according to JY/T 0587-2020. The ICSD card numbers indicating the types of calcite and quartz minerals in SL1, SL2, and L1 are 079674 and 083849, respectively [28]. The mineral content of three limestones were collected on a Rigaku Smartlab 3 kW diffractometer (Tokyo, Japan) using Cu Kα1 radiation (λ = 0.154 nm), operated in the reflection geometry (θ/2θ) at room temperature, and the X-ray tube was operated at 40 kV and 30 mA. For the sample powders, data were collected between 5° and 70°, with a scanning speed of 5°/min. Limestones SL1, SL2 and L1 were mainly composed of calcite. Siliceous limestones SL1 and SL2 contained some quartz, while limestone L1 contained a small amount of quartz. The quartz content in siliceous limestones SL1 and SL2 is 21.7 wt% and 5.4 wt%, obtained by the XRD internal standard method.

**Figure 2:**
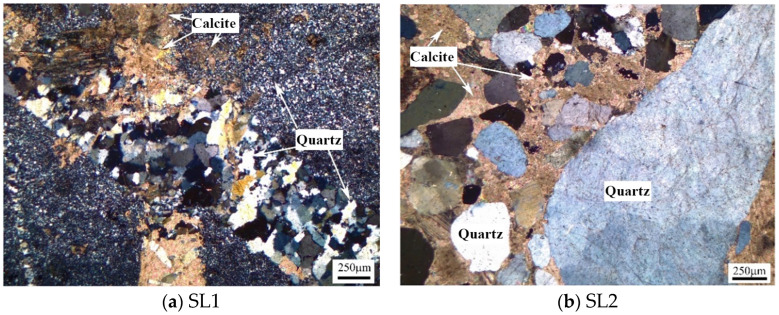
Petrographic microstructure of siliceous limestones (**a**) SL1 and (**b**) SL2.

Figure 2 demonstrates cross-polarized images of the siliceous limestones from thin sections. The morphology of quartz in SL1 and SL2 varies considerably. Siliceous limestone SL1 is mainly composed of chert nodules and calcite, and the chert nodules range from 300 to 2500 µm, composed of less than 110 µm quartz crystal. Siliceous limestone SL2 mainly consists of calcite and quartz crystals, and the crystal size of quartz in SL2 ranges from 50 to 5500 µm. 

### 2.2. Analytical Methods

#### 2.2.1. Product Fineness

Cement test ball mill SM-500 is used to produce different fineness raw meal. Siliceous limestones SL1 and SL2 are ground to 20 ± 2%, 15 ± 2%, 10 ± 2%, 7% ± 2% and 0% of residue on an 80 μm sieve, while limestone L1 is ground to 10 ± 1% of residue on an 80 μm sieve. Aluminum corrective material and iron corrective material are ground to 20 ± 1% of residue on an 80 μm sieve. Pulverized coal is calcined to coal ash and then mixed at 1.5 wt.% with other raw meal.

**Figure 3:**
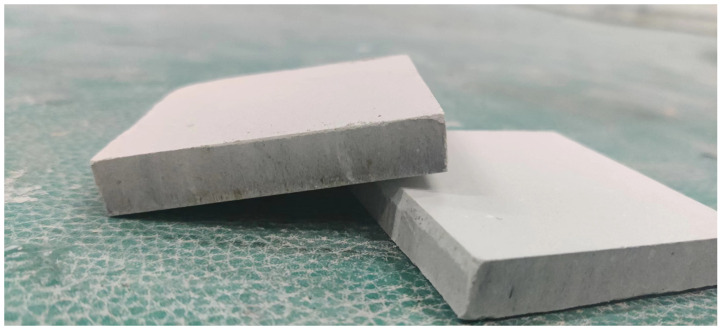
A 60 mm × 60 mm × 10 mm rectangular specimen schematic diagram for raw meal.

Based on the three modulus values of the Southwest Cement Company, the lime saturation factor (KH), silica modulus (SM), and alumina modulus (IM) are set to the following values: KH: 0.90 ± 0.02, SM: 2.20 ± 0.02 and IM: 1.50 ± 0.02. As shown in Figure 3, siliceous limestones SL1 and SL2 of different fineness were mixed with other corrective materials and pressed to form a 60 mm × 60 mm × 10 mm rectangular specimen. The raw mix design is presented in Table 2. The specimens were then thermally treated in an electric furnace at a heating rate of 5 °C/min to 900 °C and then held for 30 min, and continued to be heated at a rate of 5 °C/min to 1450 °C and held for 30 min, and were then cooled rapidly in air.

#### 2.2.2. Measurement of Quartz Content in Siliceous Limestones

**Figure 4:**
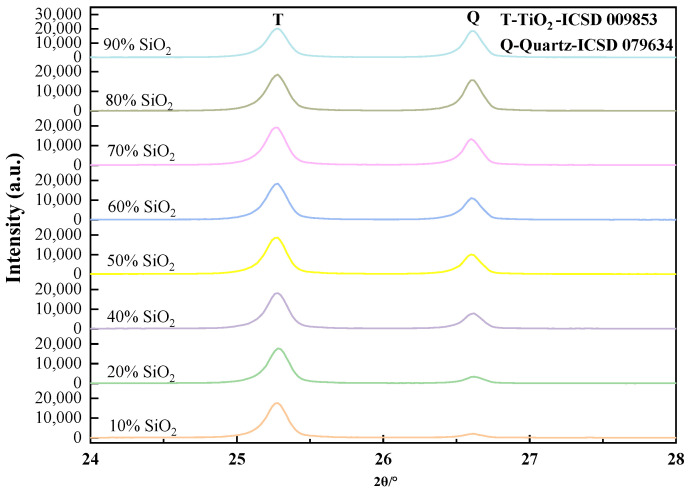
Peaks for quartz and TiO_2_ in the mixtures of Calcite-Quartz-TiO_2_.

**Figure 5:**
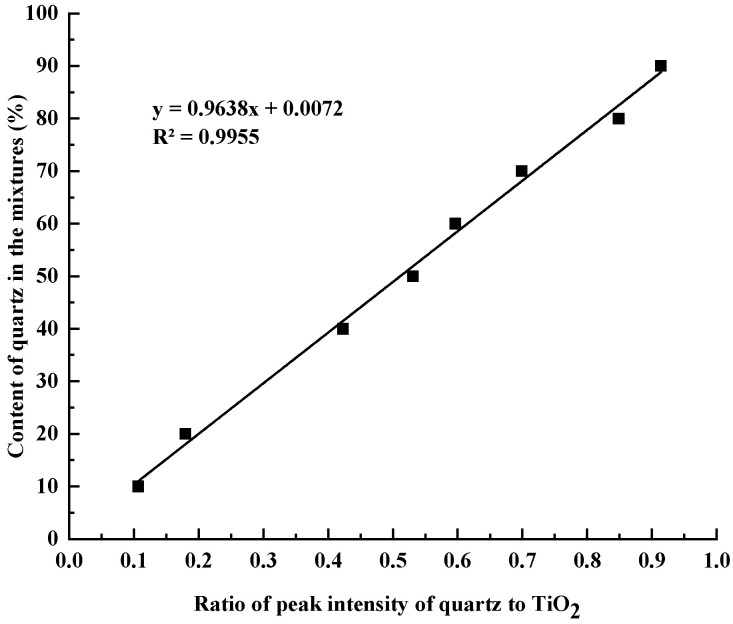
The relationship between the content of quartz and ratio of the peak intensity of quartz to TiO_2_ in the mixtures of calcite, quartz and TiO_2_.

The internal standard method of X-ray diffraction was used for the quantitative measurement of quartz in siliceous limestones. CaCO_3_ and SiO_2_, with analytical purity, were used to prepare the mixtures used for the determination of the work curve. The batch of the mixture is listed in Table 3. A total of 50% TiO_2_ with analytical purity was used as the internal standard substance, and mixed with the prepared mixtures. The X-ray powder diffraction data were collected by the Rigaku Smartlab 3kW diffractometer at 25 °C. X-ray scanning was carried out at a diffraction angle of 24° to 28° in steps of 1° per minute. The obtained data include the strongest peak of TiO_2_ and the strongest peak of SiO_2_, as shown by Figure 4. The established relationship between the content of quartz and the ratio of the peak intensity of quartz to TiO_2_ in the mixtures of calcite, quartz, and TiO_2_ is shown in Figure 5. Figure 5 presents the y-axis of the content of quartz in mixtures as a function of the (x-axis) ratio of the peak intensity of quartz and TiO_2_. A best fit linear equation and the corresponding R^2^ for the eight data points are shown in Figure 5.

Siliceous limestones with different fineness were dissolved by 1:1 dilute hydrochloric acid solution, filtered, and then dried in an oven at 105 °C for 12 h to obtain the acid insoluble content of different siliceous limestones. After that, the acid insoluble was mixed with 50% analytically pure TiO_2_ powder, and was analyzed with Rigaku Smartlab 3 kW diffractometer in the 2θ range from 24° to 28° in steps of 1° per minute. The peak intensity of quartz and TiO_2_ was calculated, and the content of quartz in siliceous limestones was determined according to Figure 5. 

#### 2.2.3. Quantification of Clinker Minerals

The calcined clinker was finely ground by a vibrating mill to pass through a 0.08 mm sieve. The free lime content in clinker was detected according to the glycol method in GB/T 176-2017. The XRD–Rietveld method was used to determine the content of C_3_S, C_2_S, C_3_A and C_4_AF, respectively. The data were collected by a Rigaku Smartlab 3 kW diffractometer from 5° to 65° (2θ) at a rate of 1°/min. The X-ray tube worked at 40 kV and 30 mA, and the quantitative analysis was performed with the Rietveld method using HighScore Plus 3.0e software. 

#### 2.2.4. Microstructure Analysis

The calcined clinker was crushed and sieved to a particle size range of 2.5–5.0 mm. The selected clinker particles were inlaid with epoxy resin and cured in an oven at 40 °C to prevent excessive epoxy from penetrating into the clinker pores. Then, the samples were finely polished with the Buehler AutoMet 250 Grinder Polisher System (Lake Country, America) to ensure that no scratches were visible on the polished surface. A 1 wt% NH_4_Cl solution was selected to etch the polished surface to make each mineral show different colors in the optical microscope. The polished surface was immersed in 1 wt% NH_4_Cl solution for 4–6 s and dried quickly with a hair dryer. An OLS4000 3D measurement laser confocal microscope (LSCM) (Tokyo, Japan) was used to observe the microstructure of the clinker phases and the distribution of the clinker minerals. Zeiss Ultra-55 field emission scanning electron microscopy (FESEM) (Jena, Germany), equipped with Oxford EDS in working condition with a 10–15 kV accelerating voltage and a vacuum environment of 1 × 10^−5^ bar, was used to analyze the elemental distribution in the clinker calcinated by siliceous limestones SL1 and SL2.

#### 2.2.5. Mechanical Property

The flexural and compressive strength of the cement clinker were tested according to the standard GB/T 17671-2021 test method of cement mortar strength [29]. The different clinkers were mixed with 5 wt.% gypsum and ground in a ball mill to form a cement with a specific surface area of 345 m^2^/kg. The mortar samples were molded to a size of 40 mm × 40 mm × 160 mm with a water to binder ratio of 0.5, and cured in a curing chamber at a relative humidity of more than 98% and a temperature of 20 ± 1 °C. The flexural and compressive strength of the specimen were measured at 28 days, and the instrument used was an ETM Series F electronic universal testing machine from Shenzhen WANCE Test Equipment Co., Ltd (Shenzhen, China).

## 3. Results

### 3.1. Quartz Distribution in Different Particle Size of Siliceous Limestones

#### 3.1.1. Content of Quartz in Different Particle Size of Siliceous Limestones

**Figure 6:**
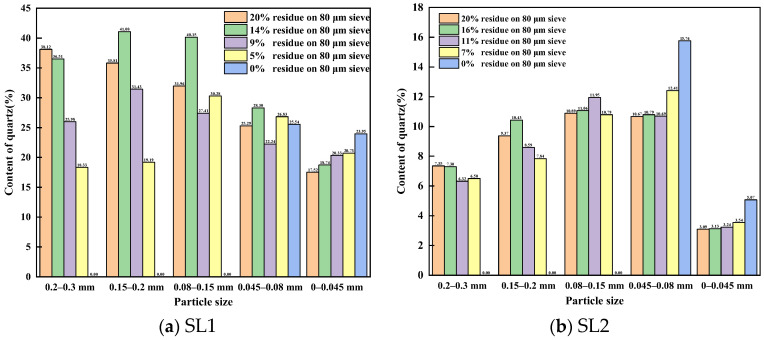
Content of quartz in different particle sizes of siliceous limestones: (**a**) SL1 and (**b**) SL2.

Based on the internal standard method, the content of quartz in siliceous limestones SL1 and SL2 were 21.7% and 5.4%, respectively. After grinding, the fineness values of siliceous limestone SL1 were 19.41%, 14.28%, 9.32%, 5.10% and 0% of residue on an 80 μm sieve, respectively. While the fineness values of SL2 were 20.22%, 16.34%, 11.39%, 6.86% and 0% of residue on an 80 μm sieve, respectively. Figure 6 shows the content of quartz in different particle sizes of siliceous limestone SL1 and SL2. It can be seen from Figure 6a,b that for different fineness values of siliceous limestone, when the particle size is large than 0.045 mm, the content of quartz in different particle size series is larger than the average quartz content in the siliceous limestone, which means large grain size quartz crystals and chert are more difficult to grind than calcite. Additionally, for particle sizes greater than 200 μm, the content of quartz continues to decrease as the fineness of the siliceous limestone decreases from 20% to 7%, while for other particle size ranges, the trend is not consistent. 

**Figure 7:**
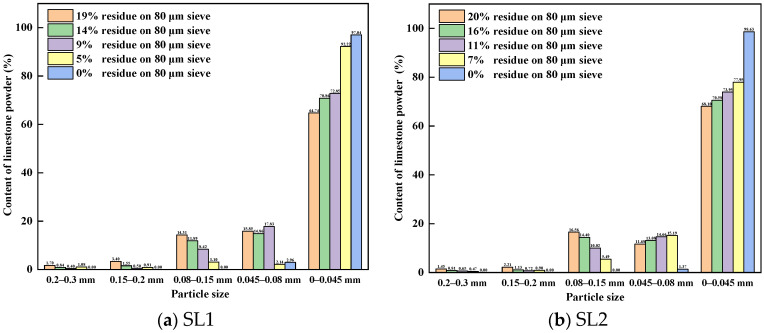
Particle size distribution of different fineness values of siliceous limestone: (**a**) SL1 and (**b**) SL2.

Figure 7 shows particle size distribution of different fineness values for siliceous limestones. It can be seen from Figure 7a,b that for siliceous limestone with different fineness values, when the particle size is greater than 0.08 mm, the powder content of each particle size gradually decreases. However, for particles with sizes between 0.045 mm and 0.08 mm, the powder content increases, indicating that with the decrease in fineness, the siliceous limestone powder is mainly concentrated below 45 µm.

**Figure 8:**
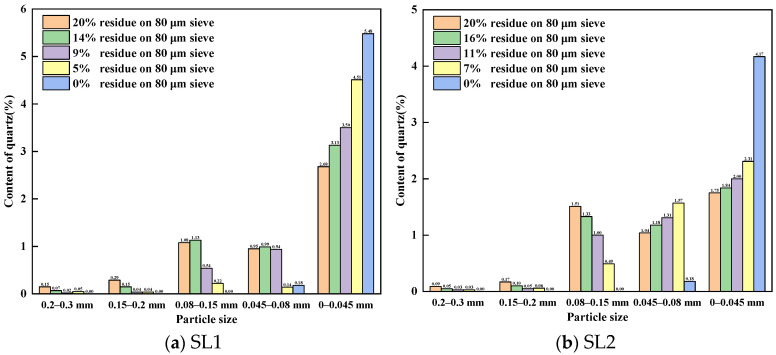
Distribution of quartz content by particle size introduced by different fineness values of siliceous limestones in the raw meal: (**a**) SL1 and (**b**) SL2.

Figure 8 shows the distribution of quartz content by the particle size introduced by different fineness values of siliceous limestones in the raw meal. As shown in Figure 8, the content of quartz decreases with decreasing particle size for siliceous limestones larger than 0.08 mm, with the exception of SL1 in the 0.08–0.15 mm range, which has a fineness of 15% on an 80 μm sieve. It is also shown that even with a fineness of 10%, there are still some quartz crystals large than 0.08 mm. However, when continuing to grind the siliceous limestone to 0% of residue on a 0.08 mm sieve, the quartz crystals are mainly located in the particle size range of 0 to 0.045 mm. Siliceous limestones SL1 and SL2 account for 96.82% and 95.86% of the quartz content in the raw material, respectively.

#### 3.1.2. Microstructure of Quartz with Different Particle Size

**Figure 9:**
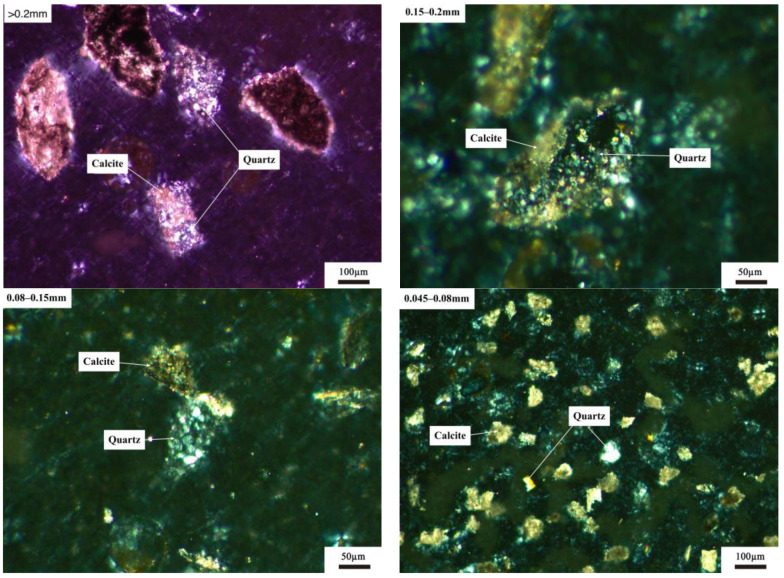
Distribution of quartz crystals with different particle sizes in siliceous limestone SL1.

The petrographic method was used to analyze the distribution of quartz crystals in siliceous limestones SL1 and SL2. Figure 9 shows the distribution of quartz with different particle sizes in SL1. As can be seen from Figure 9, quartz crystals exist independently as chert nodules in powders with a particle size greater than 0.2 mm, within 0.15–0.2 mm and within 0.045–0.080 mm. When the particle size was between 0.080 and 0.15 mm, some quartz crystals were found to be distributed in the calcite matrix, with only a small amount of quartz distributed independently.

**Figure 10:**
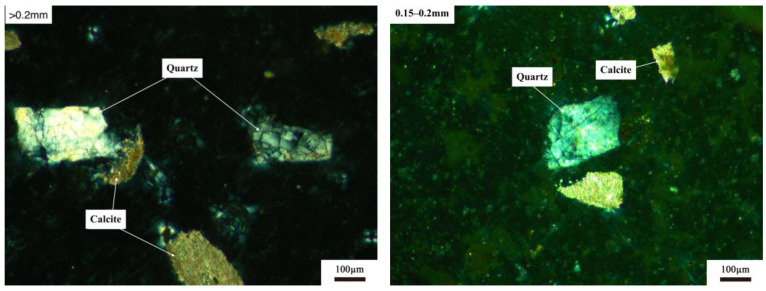

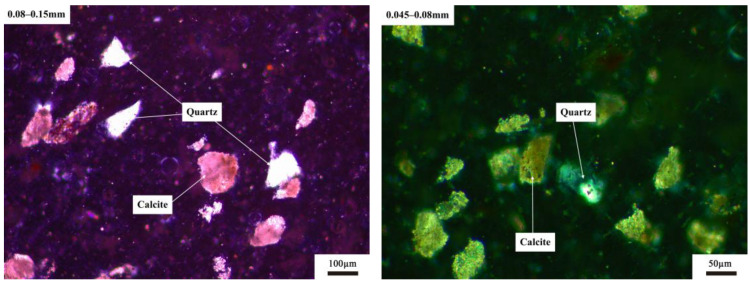
Distribution of quartz crystals with different particle sizes in siliceous limestone SL2.

Figure 10 shows the distribution of quartz with different particle sizes in SL2. As shown in Figure 10, for all particle sizes of siliceous limestone SL2, quartz crystals were found to be distributed independently in the powders, which is a little different from that of SL1. And this may due to the different morphology of quartz between SL1 and SL2.

### 3.2. Effects of Siliceous Limestone Fineness on Clinker Minerals

#### 3.2.1. Burnability and Mineral Composition

**Figure 11:**
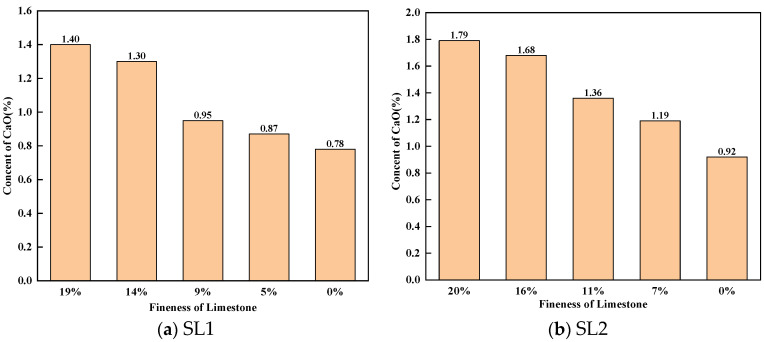
Effect of different fineness values of siliceous limestone on free lime content. (**a**) SL1 and (**b**) SL2.

Free lime content is one of the most important parameters for clinker quality. Figure 11 shows the free lime content of different clinkers calcined with siliceous limestone of different fineness. As shown in Figure 11, as the fineness of siliceous limestone in the raw meal decreases, the free lime content of clinker decreases, which indicates that the decrease in the fineness of siliceous limestone favors the burnability of clinker. With the same fineness of siliceous limestone, the free lime content of SL1 is lower than that of SL2, which indicates the burnability of SL1 is better than that of SL2.

**Figure 12:**
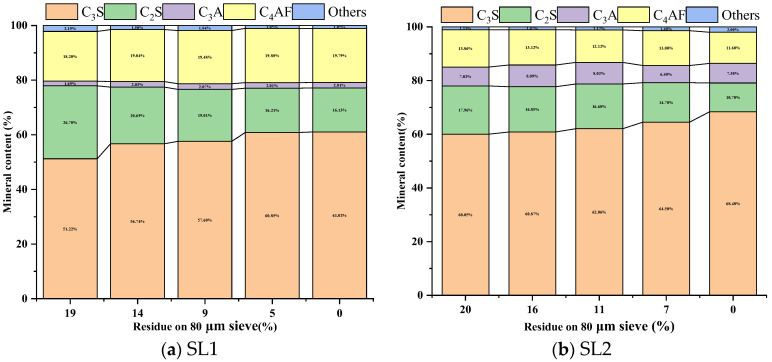
Mineral content of calcined clinker for different fineness values of siliceous limestone. (**a**) SL1 and (**b**) SL2.

Figure 12 shows the mineral content of calcined clinker for different fineness values of siliceous limestones SL1 and SL2. As shown in Figure 12a, as the fineness of the siliceous limestone decreases from 20% to 15%, the content of C_3_S increases from 51.2% to 56.7%, while the content of C_2_S decreases from 26.7% to 20.7%. However, as the fineness of SL1 decreases from 15% to 10%, the content of C_3_S only increases by 1%. It can also be seen from Figure 12a that the content of C_3_A is much lower than that of SL2. As shown in Figure 12b, with different fineness values of siliceous limestone SL2 in the raw meal, the content of C_3_S and C_2_S only vary a little. When the fineness of siliceous limestone decreases from 20% to 10%, the content of C_3_S only increases from 60% to 63%, while the content of C_2_S decreases from 18.0% to 16.7%.

#### 3.2.2. Microstructure of Clinker Minerals

**Figure 13:**
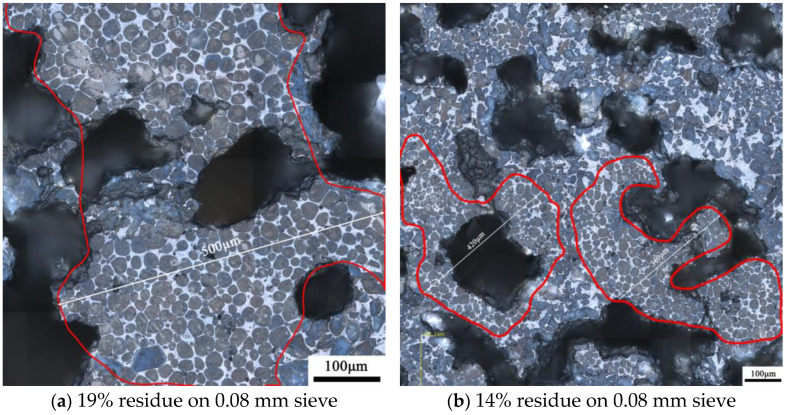

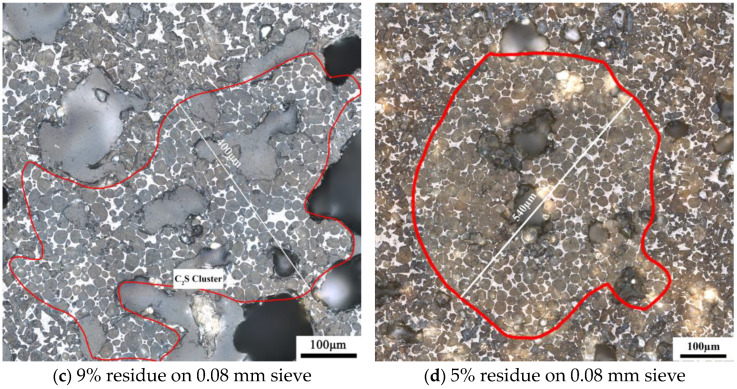
Petrographic images of clinker after calcination with different fineness values of siliceous limestone SL1. (**a**) 19% residue on 0.08 mm sieve, (**b**) 14% residue on 0.08 mm sieve, (**c**) 9% residue on 0.08 mm sieve and (**d**) 5% residue on 0.08 mm sieve.

**Figure 14:**
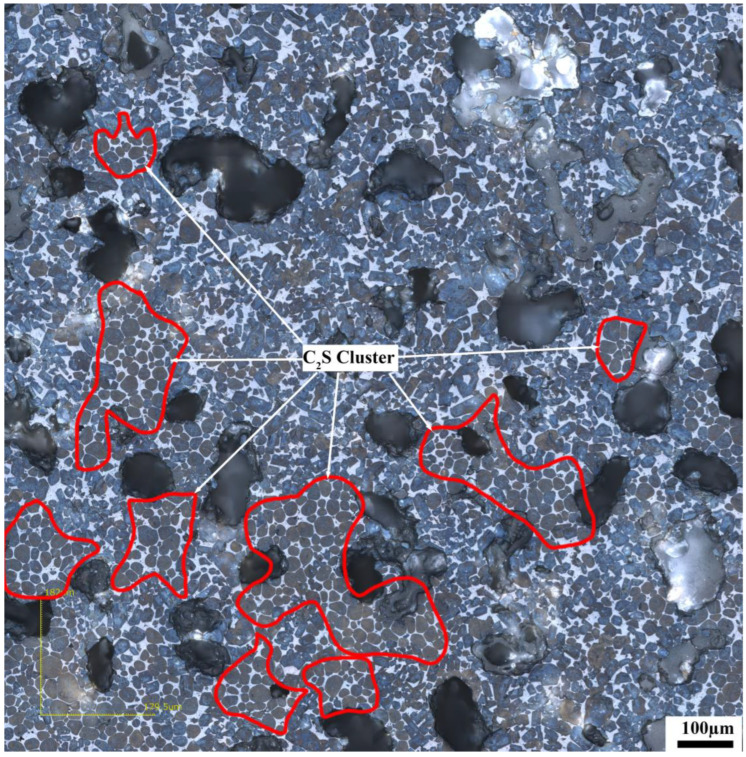
Petrographic images of clinker after calcination with siliceous limestone SL1 with a fineness of 0% of residue on 0.08 mm sieve.

Figure 13 shows the microstructure of the clinker calcined with siliceous limestone SL1 with fineness values of 19%, 14%, 9% and 5% of residue on an 0.08 mm sieve. It can be seen from Figure 13 that there are obvious C_2_S clusters in the clinker calcined with different fineness values of siliceous limestone, and most of the C_2_S clusters are within the size range of 350–550 µm. In addition, the clinker morphology does not vary much when the fineness of the siliceous limestone SL1 decreases from 19% to 5% of residue on a 0.08 mm sieve. As shown in Figure 14, when the fineness of siliceous limestone SL1 continues to decrease to 0% of residue on a 0.08 mm sieve, the size of C_2_S clusters obviously decrease, and most of the C_2_S clusters are within the size range of 50–200 µm.

**Figure 15:**
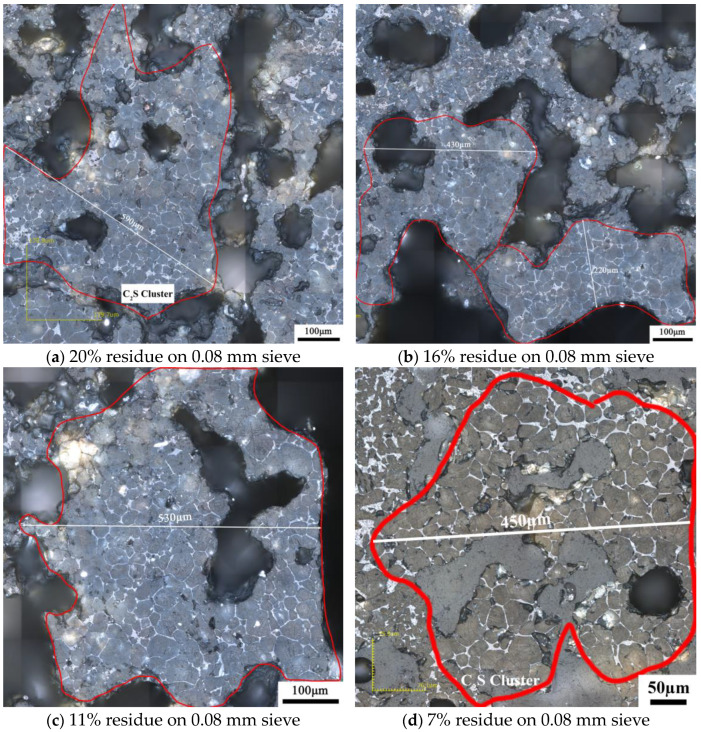
Microstructure of C_2_S cluster of different clinkers after calcination with different fineness values of siliceous limestone SL2, (**a**) 20% residue on 0.08 mm sieve, (**b**) 16% residue on 0.08 mm sieve, (**c**) 11% residue on 0.08 mm sieve and (**d**) 7% residue on 0.08 mm sieve.

**Figure 16:**
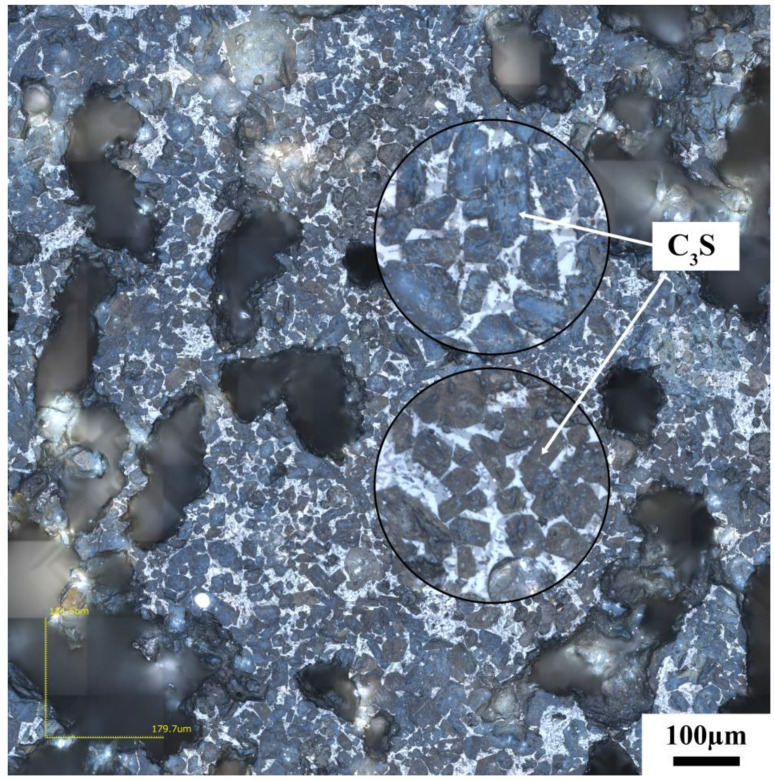
Petrographic images of clinker after calcination with siliceous limestone SL2 with a fineness of 0% of residue on 0.08 mm sieve.

Figure 15 shows the microstructure of the clinker calcined with siliceous limestone SL2 with different fineness values. Same as the clinker calcined with SL1, there are also significant C_2_S clusters for different fineness values of siliceous limestone. Most of the C_2_S clusters are within the size range of 200–600 µm, and the clinker morphology does not vary much when the fineness of the siliceous limestone SL2 decreases from 20% to 7% residue on a 0.08 mm sieve. As shown in Figure 16, when the fineness of siliceous limestone SL2 continued to decrease to 0% of residue on a 0.08 mm sieve, a little C_2_S cluster was found in the matrix of C_3_S, indicating that most of the C_2_S crystals transformed to C_3_S, with most of the quartz crystals grinded to less than 0.045 mm.

### 3.3. Effects of the Morphology of Quartz Crystal on the Microstructure of C_2_S Clusters

As shown in Figure 2, siliceous limestones SL1 and SL2 are composed of different morphologies of quartz crystal. Quartz in siliceous limestone SL1 is mainly in the form of chert nodules, while in SL2 it is mainly in the form of a single quartz crystal. Different forms of quartz in the raw meal will lead to different microstructure of C_2_S.

**Figure 17:**
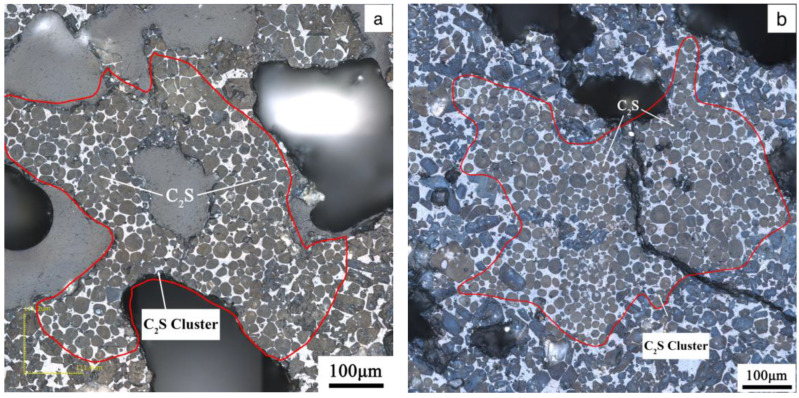
C_2_S cluster with more intermediate phase formed by calcination of chert nodule-dominated siliceous limestone SL1, (**a**) 19% residue on 0.08 mm sieve and (**b**) 9% residue on 0.08 mm sieve.

**Figure 18:**
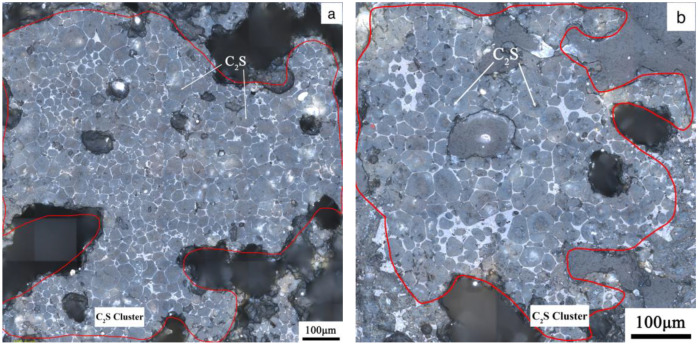
Dense C_2_S cluster with little intermediate phase formed by calcination of large grain size of quartz crystal-dominated siliceous limestone SL2, (**a**) 20% residue on 0.08 mm sieve and (**b**) 11% residue on 0.08 mm sieve.

Figure 17 shows the microstructure of a C_2_S cluster formed by the calcination of the chert nodule-dominated siliceous limestone SL1. As can be seen in Figure 17, more intermediate phases are distributed in the C_2_S cluster, except for the C_2_S clustered in a limiting region. And as shown in Figure 17b, a small amount of C_3_S can be found inside the C_2_S cluster. Figure 18 shows the microstructure of a dense C_2_S cluster formed by the calcination of large grain size of single quartz crystal-dominated siliceous limestone SL2. It can be seen from Figure 18 that there is very little of the intermediate phase inside the C_2_S cluster regions, and the shape of C_2_S is not as rounded as the traditional one. The clinker calcined by SL1 shows more intermediate phase in C_2_S cluster than that of SL2, which leads to a better burnability, and this result is consistent with the content of free lime, as shown in Figure 11. However, even though the chert module-dominated siliceous limestone will lead to more of the intermediate phase inside the C_2_S cluster, many C_2_S clusters will still be present when some large-grained chert nodules are present in the raw meal, and it will still affect the quality of the clinker.

### 3.4. Effect of Fineness of Siliceous Limestones on the Mechanical Properties of Cement

**Figure 19:**
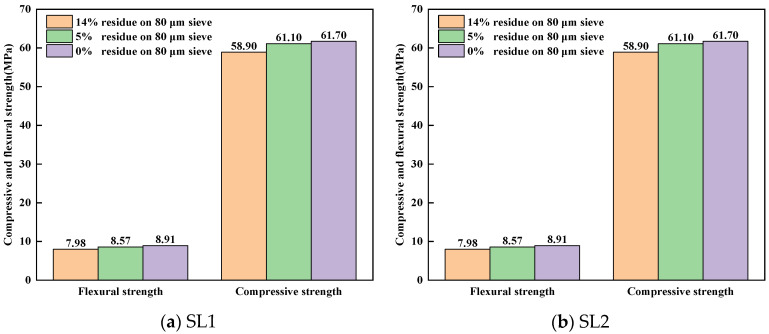
Compressive and flexural strength of the clinker calcined by different fineness values of siliceous limestone SL1 and SL2 at 28 days: (**a**) SL1 and (**b**) SL2.

Figure 19 shows the compressive and flexural strength of cement mortar samples molded with clinkers calcined by different fineness values of siliceous limestones SL1 and SL2 at 28 days. It can be seen from Figure 19 that with the decrease in the fineness of siliceous limestones SL1 and SL2, the comprehensive and flexural strength increase. When the residue on an 0.08 mm sieve is about 7%, the 28d comprehensive strength of SL1 and SL2 both exceed 60 MPa. When the residue on an 0.08 mm sieve continues to decrease to 0%, the 28d comprehensive strength of siliceous limestone SL1 and SL2 is 61.7 MPa and 62.3 MPa, respectively, which is 2.8 MPa and 4.4 MPa higher than that of clinkers calcined with 15% residue on an 0.08 mm sieve.

## 4. Discussion

The purpose of this study is to find out how quartz in siliceous limestone affects the calcination of raw meal and the quality of cement clinker. Therefore, the present discussion focuses on the explanation and limitations of the results obtained in this study.

### 4.1. Effect of Particle Size of Quartz on the Formation of Clinker Minerals

It is generally believed that coarse particles will affect the burnability of clinker, especially for quartz crystals. In our experiment, siliceous limestones were crushed and ground to a fineness of 20%, 15%, 10%, 7% and 0% of residue on an 80 µm sieve, and the content of quartz in different particle size ranges were measured. As shown in Figure 6 and Figure 8, the content of quartz in particles larger than 0.08 mm continues to decrease as the fineness of siliceous limestones decreases. However, even with a fineness of 10%, the content of quartz in particles larger than 0.08 mm is 0.60–1.08%. To produce 1 mol of C_2_S, 1 mol of SiO_2_ and 2 mol of CaO are required. The density of SiO_2_ is 2.65 g/cm^3^ and the density of C_2_S is 3.28 g/cm^3^. Thus, the volume of 1 mol of SiO_2_ is 22.67 cm^3^, while the volume of 1 mol of C_2_S is 52.46 cm^3^, which is 2.3 times than that of SiO_2_. As a result, 1 mol of SiO_2_ combined with CaO produces 1 mol of C_2_S with a volume at least 2.3 times the original volume, which does not include the intermediate phases and holes present between the C_2_S crystals. Thus, when the particle size of quartz larger than 80 µm, the size of the C_2_S cluster is at least larger than 184 µm, which is consistent with the result that most of the C_2_S clusters are within the size range of 200–600 µm. The presence of a C_2_S cluster will break the equilibrium between C_2_S and C_3_S, and as a result the C_2_S crystal in the C_2_S cluster is hard to convert to C_3_S, which leads to a reduction of C_3_S in the clinker. 

In order to minimize the drawbacks of quartz, siliceous limestones SL1 and SL2 were ground to a fineness of 0% of residue on an 80 µm sieve. The microstructure of the clinker calcinated with the ground siliceous limestones SL1 and SL2 is shown in Figure 14 and Figure 16, where the size of the C_2_S cluster decreases significantly, and even almost disappears in SL2. This means that if siliceous limestone is sufficiently fine, the adverse effects will be eliminated. However, it is unlikely that siliceous limestone can be ground that fine in plant production. For siliceous limestone SL1, when the fineness of it decreases to 5% of residue on an 80 µm sieve, the content of C_3_S in the clinker is 60.85%, and the 28d compressive strength is 61.10 MPa, which is sufficient for cement production. While for siliceous limestone SL2, when the fineness of it decreases to 7% of residue on an 80 µm sieve, the content of C_3_S in the clinker is 64.50%, and the 28d compressive strength is 60.30 MPa, which is also sufficient for cement production. This means, for different siliceous limestones, there must be an equilibrium where the amount of C_2_S clusters does not significantly affect the quality of the clinker, and the siliceous limestone does not need to be that fine. 

### 4.2. Effects of the Morphology of Quartz Crystal on the Microstructure of C_2_S Clusters

As shown in Figure 17 and Figure 18, different morphologies of quartz will lead to a different forms of C_2_S clusters. A chert nodule in raw meal may lead to a C_2_S cluster with more intermediate phase, while large-grained quartz may lead to a dense C_2_S cluster. A chert nodule is composed of microcrystalline quartz that exhibits a mosaic texture. The gap between the microcrystalline quartz may be a path for the diffusion of f-CaO and intermediate phase, thus, there is much intermediate phase inside the C_2_S cluster. As the size of the chert nodule composed of microcrystalline quartz is still too large, the C_2_S produced in the chert nodule region is still hard to convert to C_3_S in a limited calcination time.

Large-grained quartz is mainly in the form of a single quartz crystal in the raw meal, and there are few possible paths inside a single quartz crystal, through which f-CaO and intermediate phase could diffuse. This may explain the fact that little intermediate phase was found in the clinker calcinated by large-grained quartz-dominated siliceous limestone. Due to there being very little intermediate phase in the newly formed C_2_S cluster, no C_3_S was found inside the cluster, and the shape of C_2_S is not as rounded as the traditional one.

Fundal [17] highlighted the finding that a 63 µm C_2_S cluster was caused by a 44 µm quartz crystal, and then a series experiments concerning the effects of a quartz size larger than 44 µm on the burnability of raw meal were conducted. Based on Fundal’s results, Christensen [7] deduced a relationship between free lime and quartz size, and pointed out that a quartz size larger than 44 µm will have a bad effect on the burnability of raw meal. Some other researchers have also made some progress on the effects of quartz size on the burnability of raw meal [8,9,12,13,14,15,16,20], but all the results were based on the free lime content, which could not accurately reflect the quality of the clinker. In the latest study by Joseé [25], the content of different phases in the clinker calcinated by different raw meal were determined by a high-temperature X-ray diffraction. In their study, raw meal with a relatively high amount of coarse quartz (>45 µm) shows a relatively high amount of free lime and a low amount of C_3_S, which in their conclusion was due to the high content of coarse quartz in the raw meal. However, that conclusion was deduced from Fundal’s research and no other evidence was presented. While in our research, more microstructural analysis was carried out to show how different morphologies of quartz and different sizes of quartz crystal affect the quality of clinker. Besides, the microstructure of the size of C_2_S clusters and calculations of the volume of C_2_S clusters caused by quartz show that the volume of the C_2_S cluster is at least 2.3 times that of quartz. It is not appropriate to state that a quartz particle size threshold of less than 44 µm or some other size does not affect clinker quality. With the quantifications of different phases of clinker calcinated by different fineness values of raw meal and the study of the morphology of the clinker, the critical grain size of quartz varies for different types of quartz, which need further study.

### 4.3. The Reason for Low C_3_A Content in Clinker Calcined from Siliceous Limestone SL1

**Figure 20:**
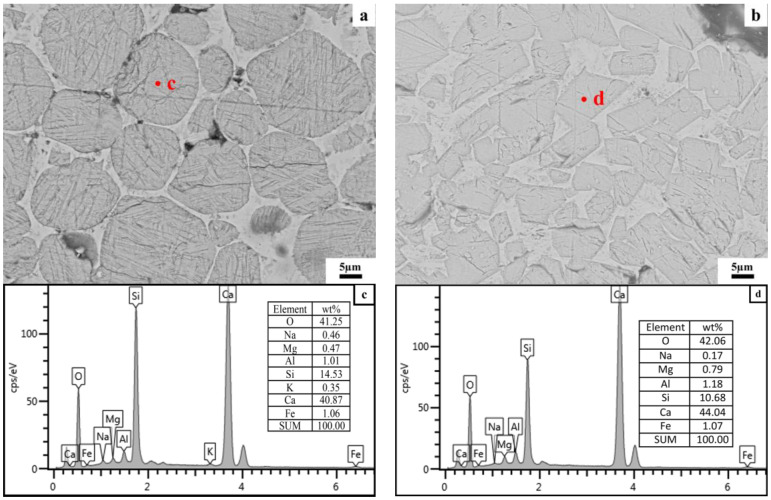
FESEM images and EDS patterns of C_2_S and C_3_S formed by calcination of siliceous limestone SL1. (**a**) C_2_S, (**b**) C_3_S, (**c**) EDS patterns for point c and (**d**) EDS patterns for point d.

**Figure 21:**
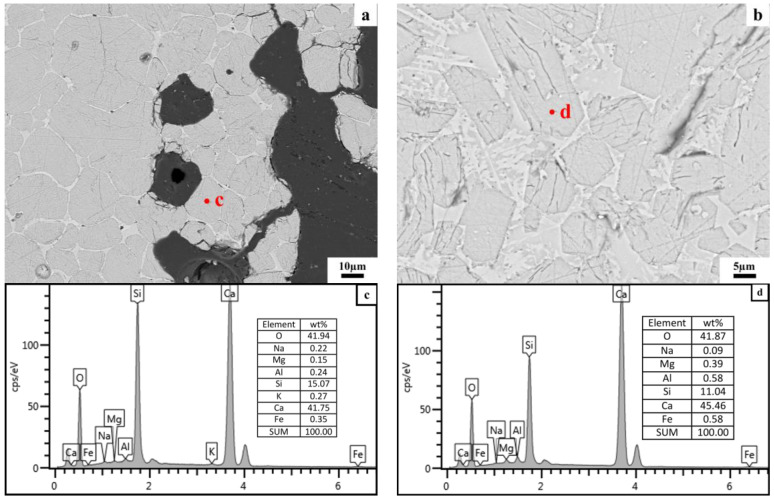
FESEM image and EDS pattern of C_2_S and C_3_S formed by calcination of siliceous limestone SL2. (**a**) C_2_S, (**b**) C_3_S, (**c**) EDS patterns for point c and (**d**) EDS patterns for point d.

In order to find out the reason for the low C_3_A content in the clinker formed by calcination of siliceous limestone SL1, FESEM equipped with EDS was used to analyze the elemental distribution of C_2_S and C_3_S in the clinker formed by SL1 and SL2. Figure 20 and Figure 21 show the FESEM images and EDS spectra of C_2_S and C_3_S formed by the calcination of siliceous limestones SL1 and SL2, respectively. As can be seen from Figure 20a,c, there exists 1.01 wt% Al in C_2_S, and the chemical formula of C_2_S based on EDS analysis is CaO_1.97_(SiO_2_) (Al_2_O_3_)_0.04_(Fe_2_O_3_)_0.02_(MgO)_0.04_(Na_2_O)_0.04_(K_2_O)_0.01_. Figure 20b,d show the FESEM image and EDS pattern of C_3_S; the content of Al is 1.18 wt% in C_3_S, and the chemical formula of C_3_S based on EDS analysis is CaO_2.89_(SiO_2_)(Al_2_O_3_)_0.06_(Fe_2_O_3_)_0.03_(MgO)_0.09_(Na_2_O)_0.01_. There is a portion of Al_2_O_3_ solidly dissolved in C_2_S and C_3_S, thus leading to the lack of C_3_A in the clinker calcinated by SL1.

Figure 21a,c show the microstructure and EDS pattern of C_2_S formed by the calcination of SL2; the content of Al is 0.24 wt% in C_2_S, and the chemical formula of C_2_S based on EDS analysis is CaO_1.94_(SiO_2_)(Al_2_O_3_)_0.01_(Fe_2_O_3_)_0.01_(MgO)_0.01_(Na_2_O)_0.01_(K_2_O)_0.01_. As shown in Figure 21 b,d, the content of Al in C_3_S is 0.58%, and the chemical formula of C_2_S based on EDS analysis is CaO_2.89_(SiO_2_)(Al_2_O_3_)_0.03_(Fe_2_O_3_)_0.01_(MgO)_0.04_. Comparing the chemical formula of the clinker formed by the calcination of SL1 and SL2, the content of Al_2_O_3_ in C_2_S and C_3_S calcinated by SL1 is higher than that of SL2, which suggests that more Al_2_O_3_ is solidly dissolved in C_2_S and C_3_S calcinated by SL1 than SL2, leading to less Al_2_O_3_ to form C_3_A, and consequently, a lower content of C_3_A in the clinker.

## 5. Conclusions

This paper assesses the effects of siliceous limestone on clinker calcination. From our experiment work, the following major conclusions can be drawn:

1. The research reveals that larger quartz crystals and chert nodules in siliceous limestone are more difficult to grind than calcite. Decreasing the fineness of siliceous limestone reduces the quantity of large quartz particles, which is beneficial for the burnability of raw meal.
2. A direct correlation between the fineness of siliceous limestone and the quality of the clinker minerals produced is noted. The fineness affects the mineral content of the clinker, which in turn affects the mechanical properties. As the fineness of siliceous limestones SL1 and SL2 decreases from 15% to 0% of residue on a 0.08 mm sieve, the content of C_3_S increases from 56.74% to 61.02% and 60.87% to 68.40%, respectively, while the content of C_2_S decreases from 20.69% to 16.13% and 16.85% to 10.70%, respectively. The compressive strength increases from 58.90 MPa to 61.70 MPa and 57.90 MPa to 62.30 MPa, respectively.
3. The structure of quartz, whether as chert nodules or single crystals, can produce C_2_S clusters during the calcination, affecting the microstructure and mineral content of the clinker. Chert nodules tend to produce C_2_S clusters with more intermediate phases, whereas large-grained single quartz crystals lead to denser C_2_S clusters with minimal intermediate phases.
4. It is important to optimize the fineness of siliceous limestone to mitigate the adverse effects of large quartz particles on clinker quality. The sufficient fineness values of siliceous limestones SL1 and SL2 are 5% and 7% of residue on a 0.08 mm sieve, respectively, which can produce a clinker with a 28d compressive strength greater than 60 MPa. Different grinding requirements are needed for the different morphologies of quartz in the raw meal, to strike a balance between clinker quality and energy consumption without having to grind siliceous limestone at very fine grades.

## Figures and Tables

**Table 1:** Chemical composition of the raw materials.

Samples	Chemical Composition/wt.%									
	LOI	SiO_2_	Fe_2_O_3_	Al_2_O_3_	CaO	MgO	K_2_O	Na_2_O	SO_3_	Total
Siliceous limestone SL1	28.33	27.02	2.16	0.48	37.00	3.80	0.17	0.07	0.07	99.10
Siliceous limestone SL2	40.08	6.48	1.08	0.24	50.91	0.24	0.16	0.05	0.07	99.31
Limestone L1	42.83	0.45	0.33	0.12	53.84	0.26	0.03	0.03	0.05	97.94
Sandstone S1	7.25	73.08	3.84	7.21	5.12	0.49	0.71	0.06	0.09	97.85
Fly ash FA	6.29	57.42	5.73	24.94	1.83	1.45	0.67	0.51	0.07	98.91
Iron slag IS	19.34	6.48	45.57	2.24	15.24	3.99	0.51	0.55	0.11	94.03
Coal ash CA	0.45	50.39	5.48	36.30	4.22	0.24	-	-	0.08	97.16

**Table 2:** Raw mix design.

No.	Raw Materials/%						
	Siliceous Limestone SL1	Siliceous Limestone SL2	Limestone L1	Sandstone S1	Fly Ash FA	Iron Slag IS	Coal Ash CA
1	-	83.5	-	2.6	7.2	5.4	1.5
2	23.6	-	61.5	-	8.3	5.1	1.5

**Table 3:** Composition of mixtures for determination of work curve.

Sample	1	2	3	4	5	6	7	8
CaCO_3_	10%	20%	40%	50%	60%	70%	80%	90%
SiO_2_	90%	80%	60%	50%	40%	30%	20%	10%
Total	100%	100%	100%	100%	100%	100%	100%	100%

## Data Availability

Data is contained within the article.
